# Effects of Hallucination Proneness and Sensory Resolution on Prior Biases in Human Perceptual Inference of Time Intervals

**DOI:** 10.1523/JNEUROSCI.0692-22.2023

**Published:** 2023-07-19

**Authors:** Emeline Duhamel, Andra Mihali, Guillermo Horga

**Affiliations:** ^1^Department of Psychiatry, Columbia University, New York State Psychiatric Institute, New York, New York 10032; ^2^Department of Psychiatry, Centre Hospitalier Universitaire de Rouen, 76031 Rouen, France

**Keywords:** hallucinations, interval timing, perceptual inference, prior beliefs, psychosis, sensory resolution

## Abstract

Bayesian models of perception posit that percepts result from the optimal integration of new sensory information and prior expectations. In turn, prominent models of perceptual disturbances in psychosis frame hallucination-like phenomena as percepts excessively biased toward perceptual prior expectations. Despite mounting support for this notion, whether this hallucination-related prior bias results secondarily from imprecise sensory representations at early processing stages or directly from alterations in perceptual priors—both suggested candidates potentially consistent with Bayesian models—remains to be tested. Using modified interval timing paradigms designed to arbitrate between these alternative hypotheses, we show in human participants (16 females and 24 males) from a nonclinical population that hallucination proneness correlates with a circumscribed form of prior bias that reflects selective differences in weighting of contextual prior variance, a prior bias that is unrelated to the effect of sensory noise and to a separate index of sensory resolution. Our results thus suggest distinct mechanisms underlying prior biases in perceptual inference and favor the notion that hallucination proneness could reflect direct alterations in the representation or use of perceptual priors independent of sensory noise.

**SIGNIFICANCE STATEMENT** Current theories of psychosis posit that hallucination proneness results from excessive influence of prior expectations on perception. It is not clear whether this prior bias represents a primary top-down process related to the representation or use of prior beliefs or instead a secondary bottom-up process stemming from imprecise sensory representations at early processing stages. To address this question, we examined interval timing behaviors captured by Bayesian perceptual-inference models. Our data support the notion that excessive influence of prior expectations associated with hallucination propensity is not directly secondary to sensory imprecision and is instead more consistent with a primary top-down process. These results help refine computational theories of psychosis and may contribute to the development of improved intervention targets.

## Introduction

An intuitive and common conception of perception is that our sensory systems accurately represent the external world as it actually is, a view that likens our perception of a visual scene to a photograph of such scene. In contrast with this view, substantial evidence indicates that perception is an idiosyncratic process that does not solely rely on input from the senses but also relies heavily on the context of what is being sensed and the expectations derived from this context. Formalizing this notion, Bayesian models of perceptual inference portray percepts as the synthesis of an optimal integration of two information sources, that is, the sensory evidence, represented by a likelihood distribution, and context-derived predictions, represented by a prior probability distribution (Stocker and Simoncelli, 2005). Here, sensory evidence is typically thought of as a bottom-up signal reflecting sensory information associated with a given incoming stimulus, whereas the context-derived prediction is a top-down signal that conveys an expectation derived from previously experienced statistical regularities among the stimuli in a given context. Critically, Bayesian models posit that these two sources of information are weighted based on their respective reliabilities; the synthesized percept will be more biased toward the prior expectation either (1) when sensory evidence is noisier (Jazayeri and Shadlen, 2010; Ryan, 2011; Cicchini et al., 2012; Karaminis et al., 2016; Egger and Jazayeri, 2018) or (2) when context-derived predictions are more precise (Friston, 2005; Stocker and Simoncelli, 2005; Zelano et al., 2011; Adams et al., 2013; Cassidy et al., 2018), and toward the mean of the sensory evidence otherwise.

Empirical data using interval reproduction and other magnitude estimation tasks support this notion. Previous interval timing studies have systematically manipulated mean interval length to induce changes in the uncertainty associated with sensory evidence (i.e., sensory noise; Lejeune and Wearden, 2009; Gu and Meck, 2011). The intuition here is that longer temporal intervals are perceived with more inherent uncertainty (i.e., one would be less certain about the exact interval duration of a longer vs a shorter interval), in line with the principle of scalar variability (Jazayeri and Shadlen, 2010; Ryan, 2011; Cicchini et al., 2012; Petzschner et al., 2015; Karaminis et al., 2016), which states that sensory noise grows linearly with interval length. Other studies have instead manipulated the width or shape of interval distributions (Miyazaki et al., 2005; Noulhiane et al., 2009; Acerbi et al., 2012; Roach et al., 2017) to induce changes in the uncertainty around prior expectations (i.e., prior variance). Consistent with Bayesian models, both manipulations (increasing mean interval length and narrowing interval distributions) have independently been shown to produce biases toward prior expectations, typically referred to in this literature as central tendency. However, to our knowledge, these two manipulations have not been applied within a single experiment to directly contrast their respective effects on perception.

Models of hallucinations and other perceptual distortions in psychosis increasingly reflect these Bayesian concepts. Although earlier views tended to conceptualize perceptual distortions as arising from imprecise or noisy bottom-up sensory signals (Javitt and Sweet, 2015), or from a failure to gate irrelevant information in these signals (Freedman et al., 1987), later views based on Bayesian models tend to emphasize overreliance on prior expectations—strong priors—as a main driver of perceptual distortions, as supported by empirical work in psychosis (Fletcher and Frith, 2009; Picard and Friston, 2014; Teufel and Nanay, 2017; Corlett et al., 2019); although other work instead supports weaker prior accounts (Weilnhammer et al., 2020), which have been often reconciled with strong prior accounts under hierarchical frameworks (Sterzer et al., 2018; Corlett et al., 2019). In addition, Bayesian views portray hallucinations as an extreme form of more common perceptual distortions, in line with the notion of a psychosis continuum from psychosis-like experiences in the general population to psychotic disorders in clinical populations (DeRosse and Karlsgodt, 2015; van Os and Reininghaus, 2016) and supported by work in subclinical psychosis-prone populations (Teufel et al., 2015; Alderson-Day et al., 2017; Powers et al., 2017; Davies et al., 2018). But despite progress in this field, the specific mechanisms that may lead to overreliance on prior expectations in psychosis remain unclear (Corlett et al., 2019). In particular, it follows from the Bayesian principle of reliability weighting that overreliance on prior expectations could arise from increased sensory noise or alterations in the representation or signaling of prior variance or a combination of both. This is relevant because it illustrates that apparently contradictory views could be reconciled in the former scenario, whereby increased sensory noise (a bottom-up signal) may secondarily produce overreliance on prior expectations (seen as a top-down signal; Teufel et al., 2015; Teufel and Nanay, 2017). Several authors (Corlett et al., 2009; Teufel et al., 2015; Horga and Abi-Dargham, 2019; Weilnhammer et al., 2020; Haarsma et al., 2022) have suggested this sensory-noise-dependent prior overweighting as a potential, even foremost (Teufel et al., 2015), candidate explanation for hallucinations, yet little empirical work has examined this explanation. In contrast, a direct alteration in the representation or signaling of prior variance, as suggested in computational models of perceptual distortions and implied by some data in psychosis (Cassidy et al., 2018), could in principle lead to over-reliance on prior expectations through a sensory-noise-independent process (Friston, 2005; Cassidy et al., 2018). Note here that evidence for the latter scenario could still be consistent with prior overreliance arising as a homeostatic adaptation to longer-term impaired sensory gain at lower processing levels as posited by hierarchical predictive coding models of psychosis (Sterzer et al., 2018); nonetheless showing that prior overreliance is not simply secondary to and commensurate with sensory noise and would indicate a prior alteration in its own right.

Here, we adapted an interval reproduction paradigm to address this question in a subclinical population of healthy individuals with varying degrees of hallucination proneness. Our paradigm parametrically manipulated both the interval mean (length) and variance (width) in a set of temporal interval distributions (conditions). This allowed us to simultaneously test for biases in the reproduced intervals toward prior expectations (i.e., central tendency) and their modulations as a function of sensory noise and prior variance, respectively. Additionally, for each subject we independently obtained a trait-like measure of sensory resolution—the Weber fraction (WF)—using standard psychophysics methods applied to a temporal categorization task (Cicchini et al., 2012). Using this design, we set out to capture distinct patterns of central tendency associated with interindividual variability in different domains (i.e., poor sensory resolution versus hallucination-like perceptual-distortion proneness). More specifically, we aimed to test whether hallucination proneness correlated with overreliance on prior expectations (i.e., increased central tendency) and whether this was explained by increased sensory noise (indexed by the Weber fraction obtained from the temporal categorization task and the length manipulation in the interval reproduction task) or primarily associated with alterations in the representation or signaling of prior uncertainty (based on the width manipulation in the interval reproduction task).

## Materials and Methods

### Subjects

Forty human participants (16 females and 24 males), recruited through advertisements posted on the Columbia University campuses, participated in this study after providing informed consent. Participants were excluded if they reported using illicit drugs in the past month or taking psychoactive medications or if they met criteria for a diagnosis of psychotic disorder based on the Structured Clinical Interview for *Diagnostic and Statistical Manual of Mental Disorders, 4th edition*, Axis I disorders, or if they reported having a neurologic disorder. This study was approved by the Institutional Review Board of the New York State Psychiatric Institute.

### Clinical and sociodemographic measures

The main measurement was the global score on the Cardiff Anomalous Perceptions Scale (CAPS; Bell et al., 2006), a widely used scale for hallucination and hallucination-like phenomena in clinical and subclinical populations. Participants were also assessed for delusion-like ideation with the Peters Delusion Inventory (PDI; Peters et al., 2004), for general cognitive function with the Letter-Number Span (LNS) working memory task (Nuechterlein et al., 2008), and with the Hollingshead scale of socioeconomic status (Hollingshead, 1975) and the Edinburgh Handedness Inventory (Oldfield, 1971).

### Experimental design and statistical analyses

#### Temporal categorization task

**Figure 1.**
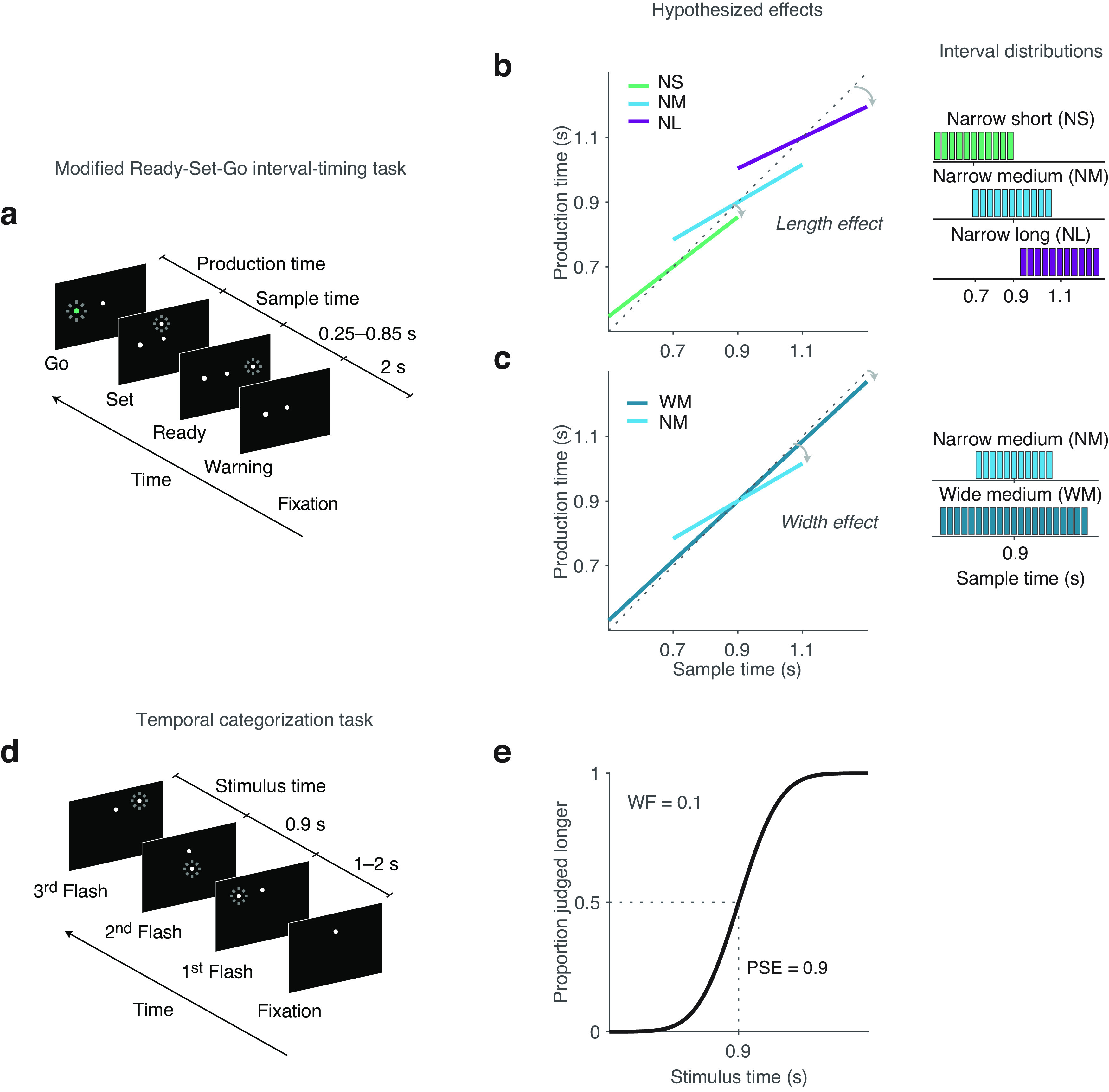
Schematic of temporal categorization and interval timing tasks. Task 1, Interval timing. ***a***, Schematic of the trial structure for the interval timing task. Subjects reproduced the sample interval (sample time) demarcated by the time between two brief flashes, Ready and Set, via a key press so that the production time was the time between the Set flash and key press. ***b***, ***c***, Four uniform prior distributions of sample intervals were used to manipulate either sensory noise by changing (***b***) the mean interval length (NS, NM, and NL) or the prior variance by changing (***c***) the interval width (NM, WM). Right, Distributions of sample intervals. In each session, sample intervals were drawn randomly from one of these distributions. Middle, Hypothesized results show increased central tendency (decreased slopes relating sample times to production times) as a function of mean interval length (length effect), indicating sensitivity to sensory noise (which increases with interval length, as expected according to the scalar variability principle) and decreased central tendency with increased interval width (width effect), indicating sensitivity to prior variance. Task 2, Temporal categorization. ***d***, Schematic of the trial structure for the temporal categorization task. Subjects performed a two-interval forced-choice task in which, after a random delay (1–2 s) following the appearance of a fixation dot, the first fixed interval was defined as the time in between two brief flashes. The second interval was defined as the time between the second and the third flashes, determined by a QUEST adaptive algorithm. Subjects then decided whether the first or the second interval was longer by pressing the corresponding key. ***e***, Example of a fitted psychometric curve derived from the temporal categorization task illustrating the two measures of interest, the PSE and the WF, an index of sensory resolution (higher WF values indicate a more shallow slope and lower resolution).

Sensory resolution of time intervals was measured with a two-interval forced-choice temporal temporal categorization task adapted from Cicchini et al. 2012, where it was referred to as a temporal bisection task (Fig. 1*d*,*e*; see Carvalho et al. (2019) for clarification on terminology). Three flashed stimuli were presented, with the time between the first and the second stimuli (interval 1) fixed at 0.9 s, and the time between the second and third stimuli (interval 2) varying based on the QUEST adaptive staircase algorithm (Watson and Pelli, 1983). Participants indicated by key press which interval was longer, 1 or 2. For training, 59 trials were presented without feedback. Of those, nine trials were drawn randomly from a uniform distribution of intervals between 0.5 and 1.3 s, which were added to facilitate fitting. Before the task session, subjects completed a training session. In the training trials, 11 intervals were randomly drawn from a uniform distribution, and feedback was provided. Subjects had to reach a performance criterion of 66% accuracy to finalize the training and proceed to the task session. On average, participants completed 1.14 training sessions. They could repeat training for a total maximum of three times. After training, participants completed 50 trials without feedback. If psychometric curve fits were poor on visual inspection, participants repeated the task up to two additional times, at which point their data for this task was excluded if fits were still poor (*n* = 1).

10.1523/JNEUROSCI.0692-22.2023.f1-1 Figure 1-1 Individual psychometric curves and model predictions for the temporal categorization task. Each subplot represents an individual participant. To visualize the psychometric curves as a function of stimulus time, we divided the stimulus time values into seven quantile bins (with about 8–9 data points each). Each black dot corresponds to a bin and represents the proportion of responses judged longer than the reference stimulus of 0.9 s. Shading indicates the SD of the model predictions for the fitted psychometric curves from a cumulative Gaussian model (see above, Materials and Methods). The fitted WF value is indicated for each subject on the top of each subplot. The pseudo-*R*-squared values (∼*R*^2^) are indicated on the bottom right of each subplot; pseudo-*R*-squared values are colored in green, except for two participants whose pseudo-*R*-squared values are at chance level based on the 99th percentile of the null distribution (in red) and for five additional participants whose values are at chance level based on the 99.9th percentile of the null distribution (orange; see above, Materials and Methods). Download Figure 1-1, EPS file.

#### Modified Ready-Set-Go interval timing task

In this task, modified from Jazayeri and Shadlen (2010; Fig. 1*a–c*), participants were instructed to measure time intervals visually demarcated by a pair of flashed stimuli and to reproduce those intervals via button press. Each trial began (Fig. 1*a*) with the presentation of a central fixation point for 2 s, followed by the presentation of a warning stimulus at a variable distance to the left of the fixation point (range of 7.5°–12.5°). After a variable delay of 0.25–0.85 s, the sample interval, *t*_s_, was measured between the first and the second flash (set flash). Participants were instructed to measure this sample interval and to reproduce it right after the presentation of the set flash by pressing the space bar. Production times, *t*_p_, were measured from the set flash to the key press. As in prior work (Jazayeri and Shadlen, 2010), when *t*_p_ fell within an adaptive target window, the warning stimulus turned green to provide positive feedback and encourage stable performance (or remained white otherwise). The target window was defined around *t*_s_ with a width scaled with the sample interval (to account for scalar timing variability) and a constant of proportionality *k*. To ensure that the performance was comparable across different prior conditions, the value of *k* was controlled by an adaptive one-up, one-down procedure that added or subtracted 0.015 to or from *k* for each miss or respective correct trial (starting at *k*_0_ = 0.1). On average, participants had 47% positive feedback, and the mean *k* value was 0.15. All stimuli were circular in shape and presented on a dark gray background. Except for the fixation point, which subtended 0.5° of visual angle, all other stimuli were 1.5°.

We parametrically manipulated the length (Fig. 1*b*) and the width (Fig. 1*c*) of the distributions from which sample intervals were drawn. We manipulated interval length using three uniform distributions with the same width (narrow) but with different interval means, the narrow-short (NS; 11 bins from 0.5 to 0.9 s, mean 0.7 s), narrow-medium (NM; 11 bins from 0.7 to 1.1 s, mean 0.9 s), and narrow-long (NL; 11 bins from 0.9 to 1.3 s, mean 1.1 s) conditions. We also manipulated interval width using two uniform distributions with the same interval mean (medium length) but different widths, the narrow-medium (as above) and wide-medium (WM; 21 bins from 0.5 to 1.3 s, mean 0.9 s) conditions. Each of these four conditions (NS, NM, NL, WM) was divided into three blocks (six repetitions per bin per block, with a 2 min rest after each block and a 10 min rest after each condition) for a total of 972 trials. To minimize variability in wide-narrow order transition effects, the order of the wide-medium condition (first vs last condition) was randomized, with the narrow conditions always appearing consecutively (first three or last three) but with their order also being randomized.

Before the task session, subjects underwent a training session, which consisted of one block of trials of the first condition with two repetitions for each bin for the wide-medium condition and four repetitions for each bin for the narrow conditions (42 and 44 trials, respectively). Subjects repeated the training up to a total of 10 times until accuracy reached 45% or higher, a criterion determined based on a binomial test. During training, subjects received positive feedback based on the adaptive target window described above. On average, subjects completed 165 training trials. The total task duration, including training, was ∼2 h.

#### Bayes least-squares model

**Figure 2.**
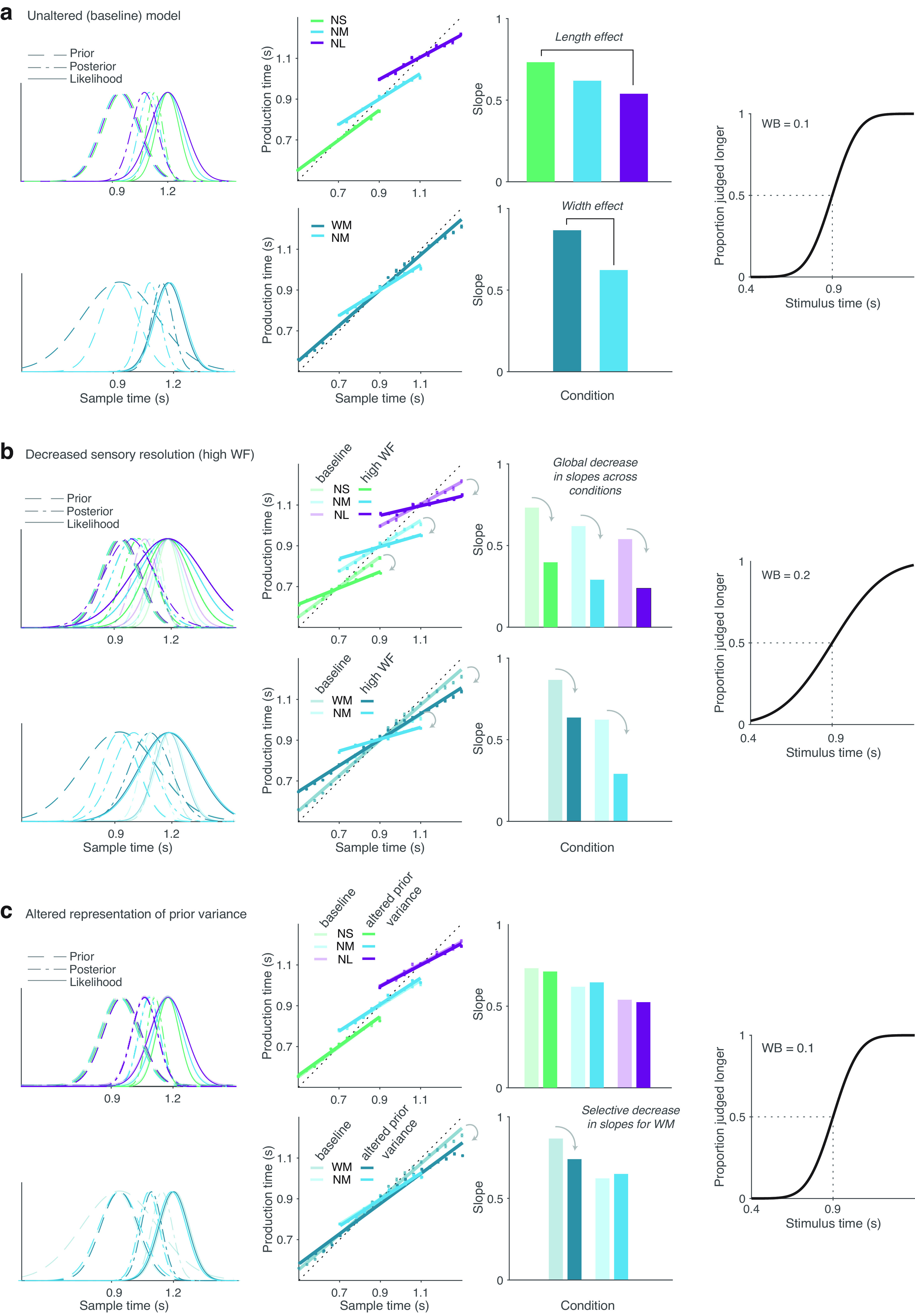
Simulations from the BLS model illustrating length and width effects on interval timing and their changes under decreased sensory resolution (higher WF) and altered representation of prior variance. ***a–c***, Baseline (unaltered) model predictions with a WF of 0.1, indicating relatively high sensory resolution representative of the average subject in our sample. Slopes, inversely proportional to central tendency, decrease with increasing mean interval length (length effect) and increase with increasing interval width (width effect), respectively, indicating increased reliance on prior expectations with increased sensory noise and with decreased prior variance. To facilitate comparisons with the baseline model predictions in ***b*** and ***c***, we show the baseline predictions of the unaltered model in lighter colors. Model predictions with decreased sensory resolution (WF = 0.2) are shown in darker colors (***b***). The central tendency values in all conditions increase (slopes decrease) relative to the baseline model in ***a***. Model predictions with constrained representation of prior variance (***c***), simulated as a reduction of the variance in the wide-medium condition; WF of 0.1 (sensory resolution as in ***a***) is shown in darker colors. The central tendency in the wide-medium condition is selectively increased (slopes decreased). The BLS model returns a posterior probability by combining the statistics of the environment as a Bayesian prior with a given likelihood (***a–c***). For illustration purposes only, the uniform distributions are transformed into Gaussian distributions (with the SD equal to the width of the uniform distribution divided by the root mean square of 12); the nonlinear output of the BLS is approximated by a linear function that intersects with the identity line at the indifference point and whose slope inversely correlates with the central tendency. Psychometric functions are cumulative Gaussians with slope representing the corresponding WF. Motor noise is set to 0.1 in all cases.

Following Jazayeri and Shadlen, 2010, we used a Bayes least-squares (BLS) model. This model integrates the statistics of the environment into a Bayesian prior, which is combined with a given likelihood to reduce overall reproduction error. To this end, a nonlinear function transforms measured variables into optimal estimates by minimizing root mean square error. This transformation introduces a bias toward the prior expectation in the estimates, consequently reducing estimation variability and overall error. We used the BLS as an ideal observer model and to predict consequences of distinct manipulations via simulations. We separately simulated the effects of increasing levels of sensory imprecision and the effects of a selective decrease in the representation of prior variance. To illustrate these effects, in Figure 2 we used a Weber fraction value of 0.2 to simulate a moderate level of sensory resolution. For illustrative purposes, the uniform distributions were converted to Gaussian distributions with the same mean and SD equal to the width of the uniform distribution divided by the root mean square of 12 (Taboga, 2021). The nonlinear BLS output was approximated by a linear function to derive robust regression lines for each condition, in line with our use of linear regression for hypothesis testing; robust regression lines were fitted to average responses from 20 simulations for each stimulus bin such that the indifference point intersected with the identity line. To simulate an alteration in the representation or signaling of prior variance in relation to hallucination proneness, which may derive from a restricted range in the neural encoding of prior variance that should mostly affect the wide-medium condition (Cicchini et al., 2012; Cassidy et al., 2018), we reduced the spread of the distribution for this condition by increasing the lower bound and decreasing the higher bound of the uniform distribution each by 10%. The simulation results were qualitatively similar regardless of the choice of parameter values.

#### Temporal categorization task analysis

Psychometric curves (Fig. 1*e*) were fitted as a cumulative Gaussian using a maximum-likelihood method (Watson, 1979). The point of subjective equality (PSE) was defined as the mean of the fitted cumulative Gaussian, and the 75% just noticeable difference (JND) was extracted from the SD of the fitted cumulative Gaussian using a scaling factor (Cicchini et al., 2012; Karaminis et al., 2016). Intuitively, the Weber–Fechner law states that the smallest difference between two stimuli that would still make them distinguishable (JND) in a discrimination experiment is proportional to the absolute magnitude of the stimulus. The WF was measured as JND/PSE. Following prior work and assuming negligible prior biases in the temporal categorization task, we interpret the WF as a trait-like individual index of sensory resolution (Karaminis et al., 2016; Hadad and Schwartz, 2019). This is because the WF determines the level of sensory noise or Bayesian likelihood variance for each interval length, where the σ_likelihood_ is often assumed to equal WF × [interval length] for a given stimulus per the scalar variability principle (Ashourian and Loewenstein, 2011; Cicchini et al., 2012; Jazayeri and Shadlen, 2010; Karaminis et al., 2016). Confidence intervals were estimated using bootstrapping.

To assess the quality of model fits, for each individual we plotted the SD of the model predictions (https://github.com/lacerbi/psybayes) to capture the uncertainty around the WF estimates (Extended Data Fig. 1-1). Following previous work (Nagelkerke, 1991; Chancel et al., 2022), we also computed a goodness-of-fit pseudo-*R*^2^ measure based on the ratio of the deviance of the cumulative Gaussian model relative to a null model of random responding (Extended Data Fig. 1-1) as implemented in the psignifit software package (Wichmann and Hill, 2001; Schütt et al., 2016). Based on simulations from the null model (500,000 iterations), we generated a null empirical distribution to assess the chance-level probability of the observed pseudo-*R*^2^ values (obtained using the cumulative Gaussian model) for each individual under the null model; values for each individual were critically above the chance level based on the 95th percentile of the null distribution (*p* < 0.05). Nonetheless, for *post hoc* analyses of robustness we also used more stringent thresholds, 99th and 99.9th percentiles, which rendered the performance of two or seven individuals, respectively, to be at chance level.

We also calculated test-retest reliability of the WF values (Hedge et al., 2018; Parsons et al., 2019). Using the odd-even splitting method (Pronk et al., 2022), the Spearman–Brown-corrected ρ (Kelley, 1925) was 0.69.

### Data censoring

To avoid artifactual results driven by distractions or extraneous events, for each individual we used the following cutoffs to discard responses that constituted obvious outliers. We first excluded trials with an error superior to 3 s (extremely unlikely because of central tendency biases, typically <0.2 s). Then, we excluded trials with absolute error above 4 SDs from the mean overall error in the task. Finally, we excluded trials with a Cook's (1977) distance above one. Following these criteria, we excluded an average of 0.59% of trials per participant.

### Linear mixed-effects model

#### Manipulation check

To validate the effects of our task manipulations, our main a priori analyses used an linear mixed-effects (LME) approach predicting reproduced intervals to test for fixed effects of central tendency [inversely correlated with the slope of the relationship between the actual intervals (i.e., the samples), and the reproduced intervals], length effects on central tendency [interaction of condition length (NL vs NS by slope)], and width effects on central tendency [interaction of condition width (WM versus NM by slope], after accounting for random intercepts and random slopes for the sample variable. Specifically, length effects were coded using dummy categorical variables for the long and medium conditions, with the short condition as reference. The length effect was then tested based on the coefficient for the long (vs short) condition variable. Similarly, width effects were coded using a dummy variable for the wide condition, with the narrow as reference, and tested based on the coefficient for the wide (vs narrow) condition variable.

In addition, we tested a number of extended LMEs to account for nonstationarity effects that we systematically observed and likely resulted from learning effects in the context of moderate numbers of trials and condition transitions within a session (Miyazaki et al., 2005; Narain et al., 2018). These nonstationarity effects included a global-mean effect (Roach et al., 2017; Lieder et al., 2019), defined by biased responses toward the mean across all previous trials; a length-transition effect (Narain et al., 2018), defined by slope shifts following transitions between narrow conditions with different interval means; a width-transition effect narrow to wide (N to W) and wide to narrow (W to N) (Miyazaki et al., 2005), defined by slope shifts following the transition between narrow and wide conditions; and a start effect, defined by slope shifts at the beginning of the first block. We compared different LMEs, from the reduced model testing our effects of interest (length and width effects on central tendency) to increasingly complex models incorporating more of the nonstationarity effects. To compare these models and in line with good practices (Wilson and Collins, 2019), we evaluated convergence across established model comparison metrics, the Akaike information criterion (AIC; Akaike, 1974), the Bayesian Information Criterion (BIC; Schwarz, 1978), and the likelihood-ratio test (LRT) for nested models (Vuong, 1989). Note that throughout all winning models were selected according to all three metrics. Because most of the nonstationarity effects tended to be restricted to the first block after a condition transition, a control analysis tested for the effects of interest using only trials from the second and third blocks (Extended Data Fig. 4-1). Finally, we evaluated multicollinearity via variance inflation factors (Hair et al., 2006), which never exceeded seven (the majority of values being smaller than five) for the reported effects of interest in winning models. Statistical significance of fixed effects used the Satterthwaite correction.

We used LME models to account for the multilevel structure of the data (Meteyard and Davies, 2020). LME models were implemented using the fitlme function in MATLAB version 2019a, assuming a normal distribution and using maximum likelihood estimation with the default optimizer (a quasi-Newton solver based on the Broyden–Fletcher–Goldfarb–Shanno algorithm). Random effects were modeled using a full covariance matrix with Cholesky parameterization. Convergence and assumptions of positive definitiveness relevant to model misspecification were checked using appropriate flags (iterative Display option for optimizer, with default tolerance value of 10^−7^, and the CheckHessian flag). These checks indicated that LME models with full random effects were generally misspecified. Instead, we used LME models including only random intercepts and random slopes for the sample variable for the following reasons: they converged appropriately, satisfied assumptions of positive definitiveness, and were always superior to both models with full random effects and models including only random intercepts according to AIC, BIC, and LRT.

LME models used in the main analyses had the following form (Wilkinson notation):

Response ∼1 + Sample * (Length + Width + Nonstationarity) + (1 + Sample|Subject),

where *Response* is the reproduced interval time, *Sample* the actual presented interval duration, *Length* and *Width* are dummy variables coded as specified above, and *Nonstationarity* represents the set of nonstationarity variables entered into a given model [absent in the simplest models, LME model 1 (LME-1), and including the entire set of variables specified above in the most complex, LME model 8 (LME-8)]. Continuous variables were *z*-scored. Within-subject random effects included the intercept and the Sample variable. Note that the principal effects of interest are the interaction terms Length×Sample and Width×Sample, which reflect modulations of central tendency by length or width, respectively (we refer to these, respectively, as “length effect” and “width effect” for simplicity). The winning model according to AIC, BIC, and LRT was LME-8, which had the following form:

Response ∼1 + Sample * (Length + Width + Start + LengthTransition + WidthTransitionNtoW + WidthTransitionWtoN) + GlobalMean + (1 + Sample|Subject).

### Tests of interindividual differences

As above, we used LMEs (including only our effects of interest or, in addition, some or all the nonstationarity effects) to test for the fixed effects of interindividual differences in sensory resolution (WF) and proneness to perceptual distortions and hallucination-like phenomena (global CAPS score) on the effects of interest; LMEs testing interindividual differences also included random intercepts and random slopes for the sample variable. These LMEs thus included six additional terms that reflected possible modulations of each of the two interindividual variables (WF and CAPS) on the sample slope (assessing central tendency), length effect, and width effect, as follows:

Response ∼1 + Sample * (Length * CAPS + Length * WF + Width * CAPS + Width * WF + Nonstationarity) + (1 + Sample|Subject).

More complex LMEs were also explored, which included interactions between nonstationarity effects and both CAPS and WF, but these LMEs showed worse goodness-of-fit indices as well as nonsignificant interactions between CAPS and each of the nonstationarity effects. As above, we compared LMEs that incorporated increasing numbers of nonstationarity effects (from LME-1 to LME-8). Continuous variables were *z*-scored. Model comparison based on AIC, BIC, and LRT selected the most complex model, LME-8, which had the following form:

Response ∼1 + Sample * (Length * CAPS + Length * WF + Width * CAPS + Width * WF + Start + LengthTransition + WidthTransitionNtoW + WidthTransitionWtoN) + GlobalMean + (1 + Sample|Subject).

Extended Data Table 4-1 contains more details on this model.

10.1523/JNEUROSCI.0692-22.2023.tab4-1 Table 4-1 The results of the winning LME model 8 from Figure 4*b*. Following Meteyard and Davies (2020), we report the fixed effects, specifically the predictors, estimates/betas, SE, confidence intervals (95%), *t* statistics and *p* values, as well as the random effects. Download Table 4-1, DOCX file.

Although our main focus in the study was to dissect the mechanisms of hallucination proneness (measured via CAPS), we included modulations with WF and CAPS simultaneously because our simulations showed that both factors could independently affect our effects of interest. We also inspected these effects in the raw data to confirm the trends of the statistical analyses and aid in the interpretation of the results, in particular the direction of interaction effects with the interindividual factors. For this, given the skewed distributions of both WF and CAPS, we plotted the data dividing groups into low and high WF (respectively, scores ≤30th and ≥70th percentile) and into low and high CAPS (respectively, scores of zero, which comprised data ≤61st percentile and ≥ 80th percentile) to visually inspect patterns of central tendency by condition in these extreme subgroups. Although other nonfrequentist hierarchical methods, like hierarchical Bayesian analyses (Haines, 2020), may be useful to probe relationships between self-reported and task-based behavioral measures, compared with frequentist regression methods they typically require stronger assumptions about priors, may provide similar results under uninformative priors (Browne and Draper, 2006; Boedeker, 2017), and are less common in the relevant literature.

## Results

Forty subjects (24 males and 16 females with mean age of 31 years) completed the study procedures (Table 1). Behavior on the temporal categorization task indicated that participants (*n* = 39 after exclusion of 1 participant; see above, Materials and Methods) accurately discriminated between temporal intervals. Fitted psychometric functions exhibited a mean point of subjective equality (PSE) of 0.84 ms (SEM = 0.003), which approximated the true stimulus mean (0.9 ms), and WF values ∼0.1 (mean = 0.12, SEM = 0.002; Table 1; Extended Data Fig. 1-1), indicating a degree of sensory resolution consistent with the prior literature and sufficient variability for examining interindividual differences (Kopec and Brody, 2010).

**Table 1.** Sociodemographic and phenomenological characteristics of the study sample (*n* = 40)

Characteristic	Descriptive statistic
Sociodemographic characteristics	
Age, mean (SEM), years	30.7 (0.23)
Sex (male/female)	24/16
Race	
Asian	11 (27%)
African American	12 (30%)
White	14 (35%)
Native American	1 (2.5%)
Other	2 (5%)
Ethnicity (Hispanic)	2 (5%)
Personal socioeconomic status, mean (SEM)	37.51 (0.45)
Parental socioeconomic status, mean (SEM)	48.31 (0.45)
Phenomenological and other characteristics	
Handedness (right-/left-handed)	36/4
CAPS global, mean (SEM), range, 0–512	19.85 (0.97), 0–163
CAPS total, mean (SEM), range, 0–480	16.95 (0.84), 0–143
PDI global, mean (SEM), range, 0–336	26.15 (0.83), 0–123
PDI total, mean (SEM), range, 0–315	23.00 (0.73), 0–110
LNS score, mean (SEM), range, 0–24	18.07 (0.08), 10–24
Weber fraction, mean (SEM)	0.12 (0.002), 0.03–0.37
Point of subjective equality, mean (SEM)	0.84 (0.003), 0.56–1.28

### Manipulations of sensory noise and of prior variance modulate central tendency

Bayesian inference schemes such as the BLS (Jazayeri and Shadlen, 2010; Petzschner et al., 2015) predict that optimal perception depends on the relative variances of both the likelihood and the prior. Consequently, the posterior distribution reflecting a percept should shift toward the prior mean as the variance in the likelihood increases and as the variance in the prior decreases. In other words, greater sensory noise or smaller prior variance should increase reliance on prior expectations, respectively, here manifesting as increased central tendency. In the context of our interval timing task, we parametrically manipulate sensory noise via changes in mean interval length (short (NS), medium (NM), long (NL); per scalar variability principle, and prior variance via changes in the width of the interval distribution (narrow, NW; wide, WM). So the above mentioned model predictions should, respectively, translate into (1) greater central tendency with longer mean interval length (Fig. 2*a*, top, reduced slopes for longer intervals), a length effect driven by sensory noise that we isolate here by contrasting the NS to the NL condition, and into (2) greater central tendency with narrower interval distributions (Fig. 2*a*, bottom, reduced slopes for narrower intervals), a width effect driven by differences in prior certainty that we isolate here by contrasting the NM to the WM condition (Fig. 2).

**Figure 3.**
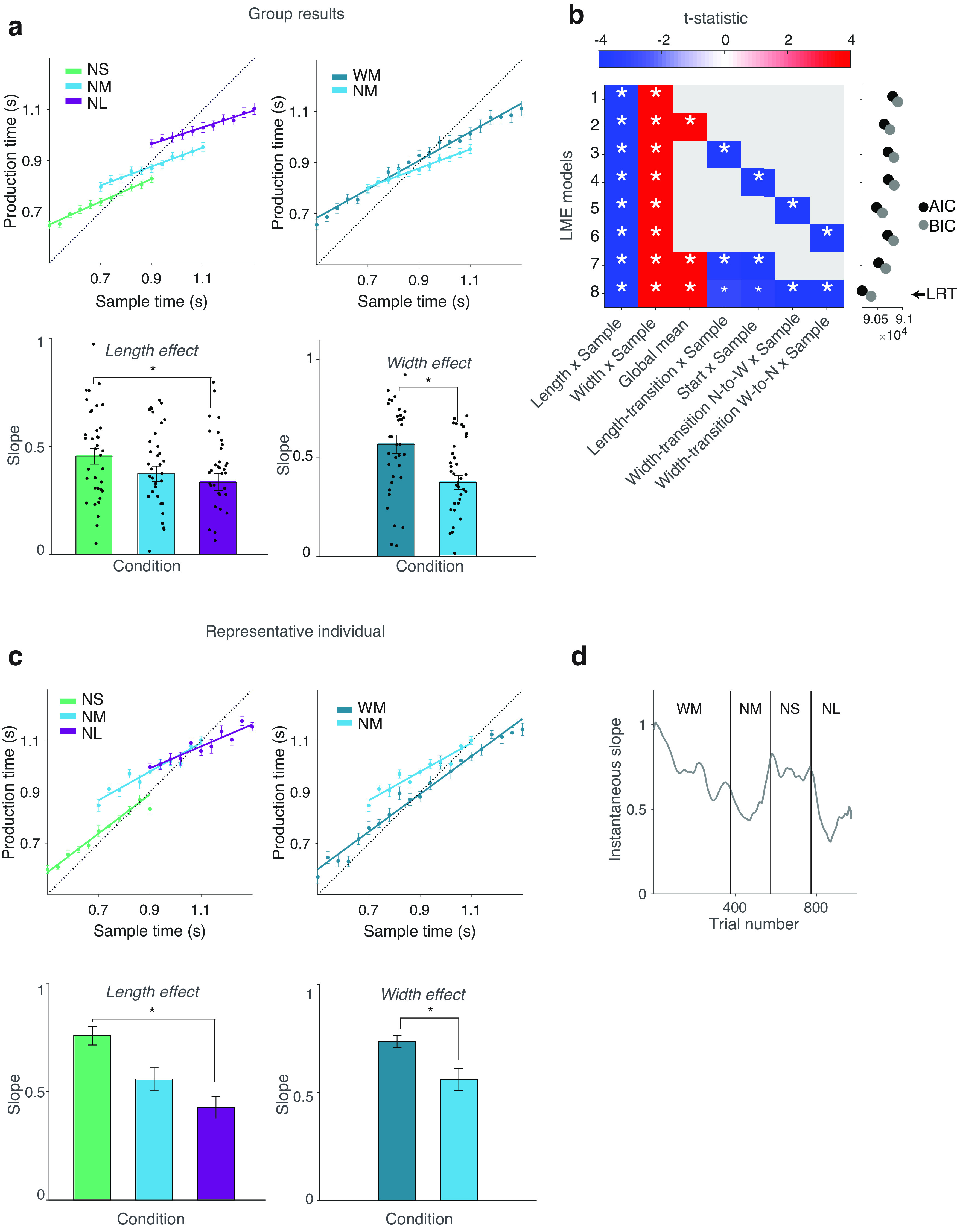
Group and individual data demonstrate length and width effects on interval timing. Top, Group-level results. ***a***, Slopes showing central tendency differences by condition (length and width effects). For visualization of results, slopes are obtained by least-squares regression of group means of mean individual responses for each bin. Error bars indicate SEM for the group for each bin. Bar plots indicate mean slopes from regression of mean individual responses for each bin. Error bars indicate SEM of the group; *p ≤ 0.05. ***b***, Matrix showing *t* statistics of fixed effects from LME for models with increasing complexity (from model 1 to model 8, with increasing number of covariates adjusting for nonstationarity effects) in the *y*-axis. Big or small white asterisks within the cells indicate statistically significant at *p* ≤ 0.001 or at *p* ≤ 0.05, respectively, for a given variable (*x*-axis). Right, Marginal plot on the right indicates AIC and BIC for each model, with the arrow indicating the winning model based on LRT, which coincides with the lowest (best) AIC and BIC. ***c***) and ***d***) Representative individual (condition order WM-NM-NS-NL, WF = 0.08, CAPS = 0). ***c***, Slopes showing central tendency differences by condition (length and width effects). Slopes are obtained by least-squares regression of mean individual responses per bin. Error bars indicate SE across responses. Bar plots show slopes and error bars indicate SEM for the regression coefficients. ***d***, Changes in central tendency over trials (instantaneous slopes) estimated from a sliding window of 43 trials in a linear regression model illustrating nonstationarity effects; instability of slopes is shown at the beginning of the task (start effect) and at transition points between conditions (indicated by vertical lines; transition effects).

The data conformed with the BLS model predictions; marked length effects and width effects were apparent in the predicted directions, both at the group level and in individual subjects (Fig. 3). On closer inspection of the data, we consistently observed several nonstationarity effects, such as condition-transition effects, in addition to the condition effects predicted by the BLS model (Fig. 3), likely because of our experimental design (see below, Discussion) and generally consistent with prior work (Miyazaki et al., 2005; Roach et al., 2017; Narain et al., 2018; Lieder et al., 2019). Importantly, the predicted length and width effects held in an extended LME [length effect (Length×Sample), *t*_(38496)_ = −4.32, *p* = 1.54 × 10^−5^; width effect (Width×Sample), *t*_(35640)_ = 9.95, *p* = 2.57 × 10^−23^, respectively] controlling for all nonstationarity effects (Fig. 3*b*, LME-8, the winning model on statistical model comparison according to AIC, BIC, and LRT; see above, Materials and Methods). This was also generally true across all simpler LMEs tested, which had less controls for nonstationarity effects but also less potential for collinearity.

### Effects of interindividual differences in sensory resolution and hallucination proneness on prior reliance

**Figure 4.**
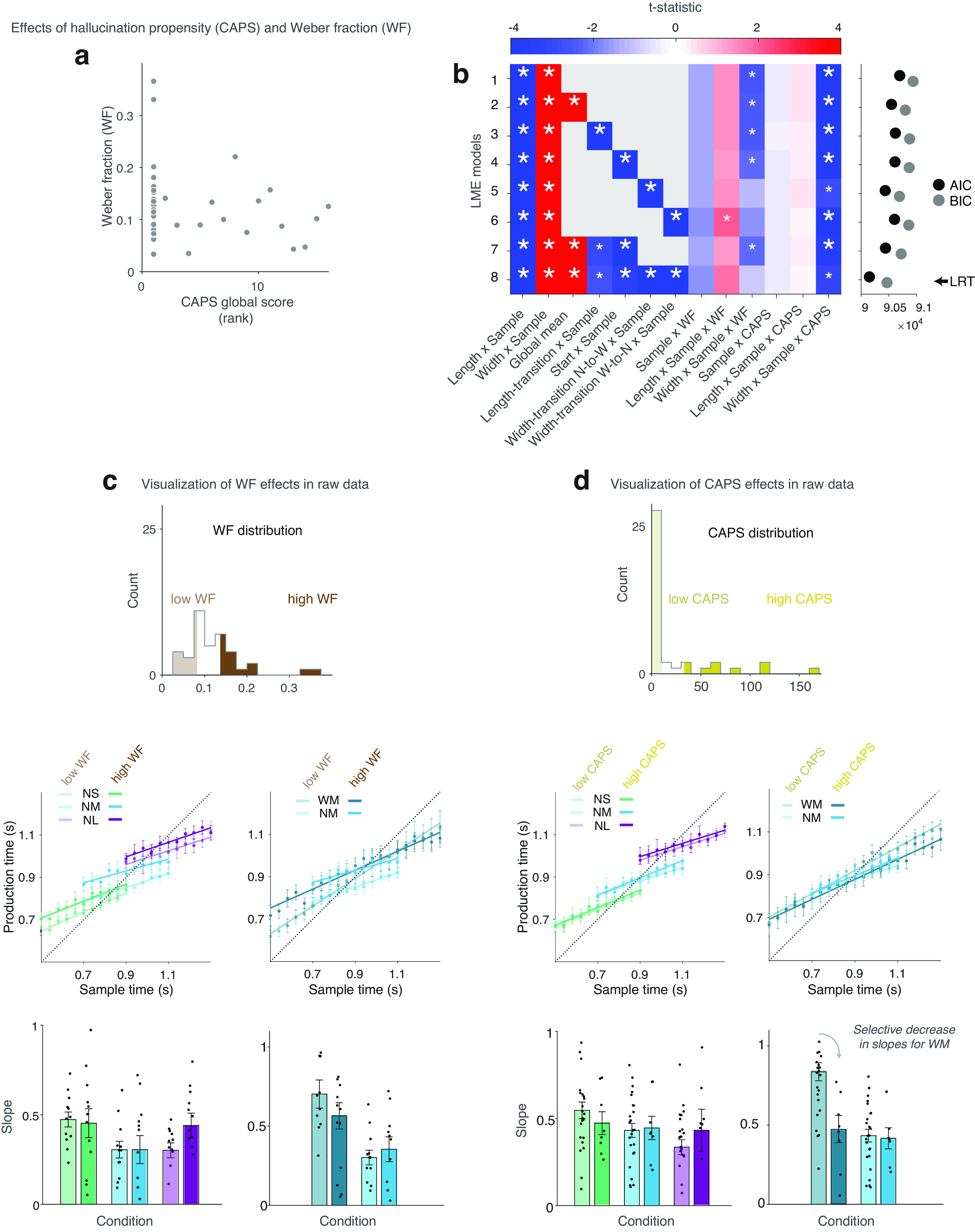
Interindividual effects of sensory resolution measured as WF, and hallucination proneness (CAPS) on central tendency, length, and width effects. ***a***, Scatter plot showing a nonsignificant relationship between CAPS scores and WF across subjects. ***b***, *t* Statistics showing fixed-effect results from LME models (conventions as in Fig. 3*a*). The winning model (model 8) is selected based on AIC, BIC, and LRT (arrow). Extended Data Fig. 4-1 depicts the *t* statistics obtained from these LME models applied to data from the last two blocks for each condition. ***c***, Mean slopes showing no significant central tendency differences by WF level (low vs high in light vs dark colors; ≤30th versus ≥70th percentile, respectively) and condition (length and width effects). ***d***, Mean adjusted slopes showing central tendency differences by CAPS level (low vs high in light vs dark colors; ≤61st or ≥80th percentile, respectively; see above, Materials and Methods) and condition (length and width effects). The significant effect of CAPS on width effects in ***b*** appears to be driven by a selective increase in central tendency (decreased slopes) in the wide-medium (WM) condition. Mean slopes are obtained by least-squares regression of group means of mean individual responses for each bin. Error bars indicate SEM for the group for each bin. For robustness, bar plots for ***c*** and ***d*** show mean of slopes from least-square regression of mean individual responses for each bin, adjusted for the variable of no interest to isolate effects of interest for each variable (i.e., for WF, slopes are adjusted by CAPS, and for CAPS, they are adjusted by WF). Error bars indicate SEM for the group.

After having shown that our key manipulations in the interval timing task induced the expected length and width effects, we examined whether interindividual variability in sensory resolution, measured as the subject's WF derived from the temporal categorization task, and hallucination proneness, measured via the scores on the CAPS self-report questionnaire, modulated these effects. Importantly, WF and CAPS scores were uncorrelated across all subjects (*r* = −0.15, *p* = 0.35; Fig. 4*a*), even in the subset with CAPS above zero (Spearman's correlation, *r* = −0.08, *p* = 0.78). This null association held in *post hoc* robustness analyses that excluded individuals with less strong evidence against random responding in the temporal categorization task (see above, Materials and Methods; Spearman's correlations after excluding two or seven participants, respectively, *r* = −0.09, *p* = 0.55 and *r* = −0.03, *p* = 0.87). These results thus suggested that hallucination proneness was independent from sensory resolution, which facilitated examining their respective interactions with effects of interest.

10.1523/JNEUROSCI.0692-22.2023.f4-1 Figure 4-1 *t* Statistics showing fixed-effect results from the LME models in Figure 4 excluding data from the first block per condition (conventions as in Fig. 4*b*). Analyses including only the last two blocks per condition. Model 5 is the winning model. Models 3, 7, and 8 could not be run because the fixed-effects design matrix was not full column rank. As expected, the start effect was no longer significant. Download Figure 4-1, EPS file.

Simulations with the BLS model served to illustrate the hypothesized effects of (1) poor sensory resolution versus (2) those of hallucination proneness; that is, (1) introducing variability or noise in the likelihood distribution through increasing WF values resulted in global increases in reliance on prior expectations, which manifested as increased central tendency (decreased slopes) across all task conditions (Fig. 2*b*) with less obvious changes in the length and width effects. In contrast, (2) simulations of a specific disruption in the representation of the prior variance, following a computational model of hallucinations and our prior work in psychosis (Cassidy et al., 2018), resulted in a qualitatively different pattern; decreasing prior variance (unsurprisingly) caused a decreased width effect driven by a selective increase of central tendency (reduced slopes) in the wide-medium condition—the condition designed to introduce the highest prior variance—with no appreciable changes in the narrow-medium condition (Fig. 2*c*).

We then tested whether interindividual variability in our empirical measures of sensory resolution (WF) and hallucination proneness (CAPS) would lead to the distinct patterns of central tendency illustrated by our simulations. To this end, our primary test consisted of an LME that allowed for modulations of individual WF and CAPS scores on overall slopes (general central tendency across conditions), the length effect, and the width effect (see above, Materials and Methods). In the winning LME (Fig. 4*b*, LME-8, according to AIC, BIC and LRT), which again incorporated all nonstationarity effects, we did not find the predicted effect of WF on central tendency (Sample×WF in LME-8, *t*_(66.8)_ = −1.65, *p* = 0.10). Critically, however, we did find the following predicted effect for CAPS; higher CAPS was associated with a marked decrease in the width effect (Width×Sample×CAPS in LME-8, *t*_(38509)_ = −3.01, *p* = 0.0026; Fig. 4). Furthermore, the modulation of the width effect by CAPS scores was consistently observed across the majority of the LME variants we evaluated (Fig. 4), indicating the robustness of this effect. The modulation of the width effect by CAPS scores was further reproduced in simple LMEs that only analyzed the last two blocks by condition (Width×Sample×CAPS in winning LME-5, *t*_(25707)_ = −3.55, *p* = 3.7 × 10^−4^; Extended Data Fig. 4-1), where nonstationarity effects were minimal, thus showing that the main result of CAPS held regardless of these nonstationarity effects and the inclusion of model covariates accounting for them. In *post hoc* robustness analyses with stringent exclusions based on WF (see above, Materials and Methods), CAPS was still associated with a reduction in the width effect (Width×Sample×CAPS in LME-8 with two excluded individuals, t_(36584)_ = −3.38, *p* = 7.3 × 10^−4^; and in LME-8 with seven excluded individuals, LME-8, t_(31778)_ = −3.99, *p* = 6.62 × 10^−5^). In contrast, no modulation of the length effect by CAPS was detected in any of the LMEs (Length×Sample×CAPS, all |*t*| values < 0.58, *p* > 0.56; Fig. 4), altogether indicating a specific involvement of prior variance but not sensory noise in hallucination proneness.

Our empirical results were thus partially consistent with model predictions; we found a distinct pattern of reliance on prior expectations as a function of hallucination proneness (CAPS) that manifested as a selective effect suggestive of altered representation of the prior variance. In contrast, sensory resolution (WF) did not exhibit an association with this selective effect or the predicted generalized increase in reliance on prior expectations. *Post hoc* analyses of specificity were inconclusive because of high collinearity between hallucination proneness scores and other psychosis proneness (e.g., PDI) scores.

## Discussion

Here, we devised an interval reproduction paradigm that can isolate the distinct influences of variability in prior expectations and sensory noise on perception of time intervals within a single experimental session. In line with Bayesian inference theory and with previous work, we observed that task conditions with longer mean intervals—and thus greater sensory noise—were accompanied by greater reliance on prior expectations (Jazayeri and Shadlen, 2010; Cicchini et al., 2012), which manifested as responses exhibiting greater central tendency. In turn, conditions with smaller prior variance were also accompanied by greater reliance on prior expectations, similarly manifesting as greater central tendency as in previous work (Miyazaki et al., 2005). Our main objective in this work, however, was attempting to identify a distinct pattern of reliance on prior expectations associated with perceptual distortions and hallucinations to arbitrate between alternative explanations of these phenomena, specifically, a model where overreliance on prior expectations depends primarily on altered representation or signaling of prior variance versus a model where this overreliance is secondary to decreased sensory resolution. To that end, we tested whether interindividual variability in two distinct perceptual domains—sensory resolution as measured via WF on a temporal categorization task and propensity for perceptual distortions and hallucination-like phenomena as measured via the CAPS self-reported questionnaire—was associated with dissociable forms of increased reliance on prior expectations. In contrast with our expectations and with previous results (Cicchini et al., 2012), we failed to detect a generalized increase in central tendency in individuals with lower sensory resolution. We nonetheless observed a distinct phenotype in individuals with higher propensity for perceptual distortions and hallucination-like phenomena consistent with our model predictions. These individuals exhibited a decreased width effect on central tendency that was seemingly driven by a selective increase in central tendency in a condition with high prior variance (i.e., the WM condition). Furthermore, hallucination propensity was uncorrelated across subjects with sensory resolution (WF) and with the sensory-noise-dependent length effect. Altogether, these findings suggest that hallucination propensity may be linked to an intrinsic and specific alteration in the neural representation or signaling of prior variance, and one that is not simply explained by sensory noise. Hallucination-prone individuals may thus fail to adequately use information about contextual variability to signal uncertainty about prior expectations and insufficiently downweigh the influence of prior expectations in this scenario; such failure leads to a pattern of overreliance on prior expectations that may be most accentuated in variable contexts that should render context-derived expectations uninformative. Our results therefore favor models of hallucinations and perceptual distortions that posit a genuine alteration in perceptual prior expectations and speak against the notion that prior overreliance necessarily results secondarily as a normative response to alterations in early sensory processes leading to sensory imprecision.

In general, our work thus provides further support for Bayesian models of time perception, building on prior work in perceptual decision-making by demonstrating simultaneous effects of manipulations in sensory noise and prior variance on central tendency, which had been previously described in separate experiments (Cicchini et al., 2012; Jazayeri and Shadlen, 2010; Miyazaki et al., 2005) and which here we were able to elicit within a single experimental session. By additionally examining interindividual variability in proneness to perceptual distortions, our current work extends these previous findings by suggesting the existence of distinct neural mechanisms governing reliance on prior expectations. In addition to a sensory-noise-dependent mechanism, our results suggest a second mechanism related to the representation or signaling of prior variance. The neural implementation of this type of mechanism may depend on multiplicative scaling of top-down expectation signals as a function of prior variance, which could be encoded in transients in neuromodulators such as dopamine or acetylcholine (Friston, 2005; Friston et al., 2012), although this has not been directly shown in interval timing tasks. Electrophysiological recordings in nonhuman primates in interval timing specifically have shown that biases in time perception may reflect biases in neuronal signals in medial prefrontal cortex and striatum (Wang et al., 2018) and may also depend on parietal cortex (Jazayeri and Shadlen, 2015). Outside interval timing tasks, human fMRI work is also compatible for a role of medial prefrontal cortex and striatum in the representation and signaling of prior variance (Behrens et al., 2007; Vilares et al., 2012). Further investigations into the neural implementation of representation or signaling of prior variance in interval timing are warranted.

Although here we evaluated individuals with subclinical proneness to perceptual distortions and hallucination-like phenomena, we believe that our findings have implications for understanding clinical hallucinations in schizophrenia. This is partly supported by evidence for the notion of a psychosis continuum and specifically by previous research showing over-reliance on prior expectations in subclinical hallucination-prone individuals (Davies et al., 2018; Powers et al., 2017; Teufel et al., 2015) as well as in hallucinating patients with schizophrenia in our own work (Cassidy et al., 2018). Specifically, our work in schizophrenia showed that patients with more severe hallucinations exhibited greater reliance on prior expectations during an interval (tone duration) reproduction task, an effect that was accentuated in conditions with high contextual variance (Cassidy et al., 2018). This hallucination-related alteration in adjustments to contextual variance (similar to the width effect in the current paradigm) further correlated with greater dopamine release capacity in the associative striatum, a well-established phenotype associated with clinical psychosis (Adams et al., 2013; Friston, 2005; Horga et al., 2014; Jardri and Denève, 2013), and with structural changes in medial prefrontal cortex, both in healthy individuals and in patients. Our current findings suggest that hallucination proneness is associated with a similar pattern of overreliance on prior expectations or strong perceptual priors, one that is most accentuated in conditions with high contextual variance (i.e., decreased width effect driven by increased central tendency in the wide-medium condition). Extending prior work, here we used a well-established psychophysics approach to derive an index of sensory resolution (i.e., WF) and tested more directly whether the hallucination-related phenotype could be secondary to poor sensory resolution or sensory imprecision, a notion that was challenged by our results—in particular the null finding of a correlation between CAPS and WF and the null modulation of CAPS on the length effect. Although this finding certainly needs confirmation in patients with schizophrenia, it supports the feasibility of models positing an alteration in the representation or signaling of prior uncertainty as a main driver of hallucinations. Furthermore, it suggests that subtle forms of these alterations are present in subclinical populations and may thus result from processes other than those associated with consequences of chronic mental illness, antipsychotic medication, and other aspects of the illness (often cited as an advantage of work in subclinical populations; Ettinger et al., 2015; Stefansson et al., 2014). Although these results do not deny the notion that strong priors may arise through a homeostatic long-term adaptation to lower-level sensory deficits (i.e., decreased gain or weaker lower-level priors; Adams et al., 2013), they do suggest that concurrent imprecision or poor resolution at the relevant sensory-input level for magnitude inferences is not necessary for and does not explain prior overreliance in relation to hallucination proneness (at least in the case of temporal interval estimation). Whether this result holds across illness stages, perceptual domains, and other hierarchical levels in more complex processes (Sterzer et al., 2018) is beyond the scope of this work and warrants future investigation.

Interestingly, the phenotype we describe in relation to hallucination proneness stands in contrast with that reported in individuals with autistic spectrum disorder with a similar approach to ours (Karaminis et al., 2016). Individuals with autism exhibited increased poor sensory resolution together with proportionally insufficient reliance on prior expectations, an alteration that could lead to inadequate reliance on noisy sensory evidence and that is reminiscent of sensory-gating models. This phenotype is distinct from—almost opposite to—the one we observed in hallucination-prone individuals, who exhibited spared sensory resolution (WF) and sensitivity to sensory noise (length effect) and disproportionate reliance on prior expectations, although the latter was most evident in conditions with high prior variance that were not probed in the previous autism work.

Some limitations of our work are worth discussing. Because of our interest in psychosis, we set out to develop a paradigm that would be usable in clinical populations, which necessarily limited the duration of the experiment. Our paradigm thus featured fewer trials than previous work and more transitions between conditions within the experimental session. This is likely the main reason for the nonstationarity effects we observed, which have been previously documented but are not captured by static models such as the BLS. Although this is a limitation that here we attempted to address by modeling nonstationarity effects within a LME framework, future work should capitalize on the rich set of nonstationarity effects we describe to derive trial-by-trial learning models of interval timing capable of accounting for both BLS-predicted and nonstationarity effects (Narain et al., 2018). This work should specifically attempt to address a limitation of our approach in separating acquisition versus use of prior expectations and examine their respective contributions to hallucinations. One potential direction would be to extend existing biophysical models of interval timing, which already capture some condition-transition effects (Narain et al., 2018). Relatedly, one interesting future direction would be to examine trial-by-trial acquisition of prior beliefs in relation to nonstationarity effects and hallucinations, although preliminary analyses did not detect interactions between CAPS and any of the nonstationarity effects. Models wherein sensory noise in relation to interval length could induce distorted prior representations should also be considered, even if such distortions (associated with the length manipulation) may be small relative to the direct manipulation of prior variance (i.e., the width manipulation) and considering the null effect of CAPS on length effects. Future work should aim to enhance reliability of sensory resolution measures, perhaps increasing the number of trials. Although here we aimed to dissect a hallucination-related mechanism for overreliance on prior expectations using an interval timing paradigm, we cannot conclude that a similar alteration is present across other sensory modalities. This should be a focus of future work. Nonetheless, similar hallucination-related phenotypes have been observed across different modalities (Corlett et al., 2019; Horga et al., 2014; Powers et al., 2017), potentially suggesting a supramodal alteration in top-down processes.

In conclusion, by adapting an interval reproduction paradigm and using established psychophysics methods, we found in a subclinical population that overreliance on prior expectations in relation to hallucination propensity was primarily related to altered representation or signaling of prior variance, rather than secondary to increased sensory imprecision. Our work directly implicates alterations in prior beliefs in hallucination proneness and provides new tools for necessary empirical research to refine current conceptualizations of psychosis.
