# Supplementary material for: Effects of Hallucination Proneness and Sensory Resolution on Prior Biases in Human Perceptual Inference of Time Intervals
Source: J Neurosci. 2023 Jul 19;43(29):5365–77. doi: 10.1523/JNEUROSCI.0692-22.2023 (PMC10359030; doi:10.1523/JNEUROSCI.0692-22.2023)
Supplement: Table 4-1 — The results of the winning LME model 8 from Figure 4b. Following Meteyard and Davies (2020), we report the fixed effects, specifically the predictors, estimates/betas, SE, confidence intervals (95%), t statistics and p values, as well as the random effects. Download Table 4-1, DOCX file. [file ns-JN-RM-0692-22-s03.docx]

| **Winning LME (LME-8) including interactions with CAPS and WF (Fig. 4b)** | | | | | |
| --- | --- | --- | --- | --- | --- |
| **Fixed Effects** | | | | | |
|  | Est/Beta | SE | 95% CI | t | p |
| Intercept | -0.24 | 0.05 | -0.34 – -0.15 | -4.92 | 8.53E-07 |
| Sample | 0.39 | 0.03 | 0.33 – 0.45 | 12.52 | 6.7922E-36 |
| Length S to M | 0.17 | 0.02 | 0.13 – 0.21 | 9.18 | 4.7114E-20 |
| Length S to L | 0.53 | 0.02 | 0.49 – 0.58 | 22.53 | 1.1175E-111 |
| Width | 0.25 | 0.02 | 0.22 – 0.28 | 15.71 | 1.9229E-55 |
| Width-transition  N-to-W | -0.25 | 0.02 | -0.28 – -0.21 | -13.30 | 2.8397E-40 |
| CAPS | -0.01 | 0.05 | -0.11 – 0.09 | -0.20 | 0.83983 |
| WF | 0.05 | 0.05 | -0.05 – 0.15 | 0.96 | 0.3359 |
| Global Mean | 0.04 | 0.01 | 0.03 – 0.06 | 6.99 | 2.7607E-12 |
| Length-transition | -0.04 | 0.01 | -0.06 – -0.02 | -3.28 | 0.0010501 |
| Width-transition W-to-N | 0.14 | 0.02 | 0.11 – 0.18 | 8.38 | 5.5225E-17 |
| Length (S to M) × Sample | -0.05 | 0.02 | -0.09 – -0.01 | -2.36 | 0.018362 |
| Length × Sample | -0.09 | 0.02 | -0.14 – -0.05 | -4.34 | 1.44E-5 |
| Width × Sample | 0.19 | 0.02 | 0.15 – 0.23 | 9.28 | 1.782E-20 |
| Width-transition  N-to-W × Sample | -0.13 | 0.02 | -0.17 – -0.09 | -7.18 | 7.0834E-13 |
| Sample × CAPS | -0.01 | 0.03 | -0.08 – 0.05 | -0.45 | 0.65308 |
| Length (S to M) × CAPS | 0.02 | 0.02 | -0.02 – 0.06 | 1.13 | 0.25718 |
| Length × CAPS | 0.03 | 0.02 | -0.01 – 0.08 | 1.49 | 0.1373 |
| Width × CAPS | -0.03 | 0.01 | -0.05 – -0.01 | -2.57 | 0.010292 |
| Sample × WF | -0.05 | 0.03 | -0.11 – 0.01 | -1.65 | 0.099771 |
| Length (S to M) × WF | 0.03 | 0.02 | 0.00 – 0.07 | 1.76 | 0.078013 |
| Length × WF | 0.04 | 0.02 | -0.01 – 0.09 | 1.68 | 0.093049 |
| Width × WF | 0.02 | 0.01 | 0.00 – 0.04 | 1.73 | 0.083327 |
| Length-transition × Sample | -0.03 | 0.01 | -0.06 – -0.01 | -2.67 | 0.0076121 |
| Width-transition W-to-N × Sample | -0.09 | 0.02 | -0.13 – -0.05 | -4.73 | 0.0000023 |
| Start | 0.12 | 0.01 | 0.10 – 0.15 | 9.21 | 3.3961E-20 |
| Length (S to M) × Sample × CAPS | 0.03 | 0.02 | -0.01 – 0.07 | 1.45 | 0.1458 |
| Length × Sample × CAPS | 0.00 | 0.02 | -0.04 – 0.05 | 0.18 | 0.86053 |
| Width × Sample × CAPS | -0.05 | 0.02 | -0.08 – -0.02 | -3.01 | 0.0025807 |
| Length (S to M) × Sample × WF | 0.01 | 0.02 | -0.03 – 0.05 | 0.54 | 0.58827 |
| Length × Sample × WF | 0.04 | 0.02 | 0.00 – 0.08 | 1.78 | 0.075161 |
| Width × Sample × WF | -0.02 | 0.02 | -0.05 – 0.02 | -0.94 | 0.34969 |
| Start × Sample | -0.04 | 0.01 | -0.07 – -0.02 | -3.29 | 0.00099987 |
|  | | | | | |
| **Random Effects** | | | | | |
|  | | | Variance | SD | Correlation |
| Sample \| Participant (Intercept) | | | 0.085 | 0.291 |  |
| Sample (slope) | | | 0.029 | 0.171 | 0.328 |
|  | | | | | |
| **Model Fit** | | | | | |
| R^2^ | | | Marginal | Conditional | |
|  | | | 0.287 | 0.3998 | |
| **Key:** p-values for fixed effects calculated using the Satterthwaite approximations.  **Model equation** (Wilkinson notation)**:** Response ~ 1 + Global Mean + Sample * (Length * CAPS + Length * WF + Width * CAPS + Width * WF + Length-transition + Start + Width-transition N-to-W + Width-transition W-to-N) + (1 + Sample \| Participant) | | | | | |
